# Materia Medica

**Published:** 1838-07

**Authors:** 


### MATERIA MEDICA.

### *New Preparation of Ipecacuanha.*

M. Gay describes a new mode of preparing this medicine, which may have its advantages in certain cases. The following is the formula:

| | | |
|---|---|---|
| Ipecacuanha, in powder | . . | 1 part. |
| Rectified Sulphuric Æther | . | 6 parts. |

Macerate for some hours, and filter. Dry, by exposure to the air, the powder remaining upon the filter till it has entirely lost the odour of æther; then triturate gently, and preserve for use.—Ipecacuanha thus prepared is administered in the same doses as ordinary ipecacuanha, having all the properties of the latter: it has only lost its nauseous odour and disagreeable taste.

*Bulletin Général de Thérapeutique. August,* 1837.

